# Classification of the *Pospiviroidae* based on their structural hallmarks

**DOI:** 10.1371/journal.pone.0182536

**Published:** 2017-08-04

**Authors:** Tamara Giguère, Jean-Pierre Perreault

**Affiliations:** RNA Group/Groupe ARN, Département de biochimie, Faculté de médecine et des sciences de la santé, Pavillon de recherche appliquée sur le cancer, Université de Sherbrooke, Sherbrooke, Québec, Canada; Oklahoma State University, UNITED STATES

## Abstract

The simplest known plant pathogens are the viroids. Because of their non-coding single-stranded circular RNA genome, they depend on both their sequence and their structure for both a successful infection and their replication. In the recent years, important progress in the elucidation of their structures was achieved using an adaptation of the selective 2’-hydroxyl acylation analyzed by primer extension (SHAPE) protocol in order to probe viroid structures in solution. Previously, SHAPE has been adapted to elucidate the structures of all of the members of the family *Avsunviroidae*, as well as those of a few members of the family *Pospiviroidae*. In this study, with the goal of providing an entire compendium of the secondary structures of the various viroid species, a total of thirteen new *Pospiviroidae* members were probed in solution using the SHAPE protocol. More specifically, the secondary structures of eleven species for which the genus was previously known were initially elucidated. At this point, considering all of the SHAPE elucidated secondary structures, a classification system for viroids in their respective genera was proposed. On the basis of the structural classification reported here, the probings of both the *Grapevine latent viroid* and the *Dahlia latent viroid* provide sound arguments for the determination of their respective genera, which appear to be *Apscaviroid* and *Hostuviroid*, respectively. More importantly, this study provides the complete repertoire of the secondary structures, mapped in solution, of all of the accepted viroid species reported thus far. In addition, a classification scheme based on structural hallmarks, an important tool for many biological studies, is proposed.

## Introduction

Viroids are plant pathogens found in many agriculturally important cultures [1]. They are simple pathogens composed of a single-stranded circular, non-coding RNA genome of 246 to 401 nucleotides (nt). To date, a total of thirty-two distinct species are generally accepted as existing by the scientific community, all of which can be classified into two families [2]. The first of these, the *Avsunviroidae*, is a small group composed of four species. All possess a hammerhead self-cleaving motif and replicate in the chloroplast through a symmetrical rolling circle mechanism. The type species of this family is the *Avocado sunblotch viroid*. The second, the *Pospiviroidae*, includes twenty-eight members that replicate in the nucleus via an asymmetric rolling circle mechanism. Most of the members of the *Pospiviroidae* fold into a rod-like structure which includes five regions: the terminal left (TL), the pathogenic (P), the central (C), the variable (V) and the terminal right (TR). Furthermore, the members of the family *Pospiviroidae* are categorized, based on the sequence identity in their central conserved region (CCR) and/or their biological features, such as known hosts and symptoms, into five genera: *Pospiviroid*, *Hostuviroid*, *Apscaviroid*, *Cocadviroid* and *Coleviroid*.

The secondary structure of a viroid is of primary importance for its interactions with the host’s cellular components. During its life cycle, the viroid must move from one cellular compartment to another, following interaction with the host’s proteins, through the use of either RNA motifs or of its folded structure [3]. Its nucleotide sequence is also crucial, as it has been shown to interact with the host genome, for example as in the methylation of the DNA [4], or with the host RNA in the form of viroid derived small RNA (vd-sRNA) in post-transcriptional gene silencing (PTGS) [5].

Until recently, most viroid structures were obtained by computer prediction and, consequently, were imprecise. Biochemical approaches were not used as they were simply too tedious to be performed. Hence, the technique of the selective 2’-hydroxyl (2’-OH) acylation analyzed by primer extension (SHAPE) was adapted to the study of viroid structures [6]. Briefly, the technique uses an electrophilic reagent that reacts with the 2’-OH group of the ribose residues of the RNA when it is located in a flexible conformation, that is to say when it is single-stranded, forming a chemical adduct on the nucleotide [6]. During the primer extension step that follows this chemical reaction, the reverse transcriptase is unable to pass through the adduct, and thus produces cDNA fragments whose lengths correspond to the flexibility of the specific nucleotides of the RNA. The positions of those nucleotides, and the strengths of their signals (radioactive or fluorescent), are revealed by electrophoresis. The normalized biochemical data obtained by SHAPE are then computed as pseudo-free energy terms, and are used in the energy function of the structure prediction software, thus generating a more accurate structure.

Initially, it was shown that the SHAPE-based structure of the *Peach latent mosaic viroid* (PLMVd) of (+) polarity was in good agreement with that previously obtained from classical enzymatic probing [7–9]. Subsequently, the probing of the (+) strands of several PLMVd variants led to the identification of new RNA motifs, including new pseudoknot and cruciform structures, illustrating that viroids are heterogeneous not only at the sequence level, but also at the structural level [7–10]. Subsequently, the structures of the strands of both polarities of the four members of the family *Avsunviroidae*, as well as that of non-confirmed member (specifically the *Grapevine hammerhead viroid-like*), were elucidated [9]. Other reports have used SHAPE to study different variants of *Avsunviroidae* such as the *Avocado sunblotch viroid* (ASBVd) [11] and the *Eggplant latent viroid* (ELVd) [12].

The structures of seventeen members of the family *Pospiviroidae* belonging to several genera were also resolved although this list remains incomplete so far [13,14]. Interestingly, another group also probed a different sequence variant of *Potato spindle tuber viroid* (PSTVd) using the same approach [15]. The resulting *in vitro* structure was relatively similar to those proposed previously although it also showed local structural differences [5,14]. The *in vivo* SHAPE probing obtained for the PSTVd variant was in good agreement with the *in vitro* SHAPE structure, except for the fact that the former appeared to be slightly more reactive towards the SHAPE reagent [15]. This suggested that the presence of any potential RNA binding proteins did not seem to significantly interfere with the viroid’s structure [15]. In brief, these studies provide physical evidence that the prediction of a secondary structure based on *in vitro* SHAPE probing is relevant. Moreover, the previous mapping in solution of the structures of several viroid species established that an average of 22% of the nucleotides were not located in the same pairing situation in the structure folded using the SHAPE data as compared to that obtained without using it [14]. Consequently, it is suggested that SHAPE probing should be the gold standard with which to determine a viroid’s secondary structure, which can then be further used as a model for its biological characterization.

In this report, the aforementioned structure list was completed by elucidating the structure of thirteen additional *Pospiviroidae* members so as to cover those of all viroid species. First, the secondary structures of eleven species that had previously been assigned to a specific genus were elucidated. Taking into consideration all of the established structures at this point, a classification of all genera was deduced based on structural hallmarks. Subsequently, the SHAPE probings and structural predictions of the novel viroids, *Grapevine latent viroid* and *Dahlia latent viroid* were performed, and their respective genera were deduced. Altogether, this study and the previous ones provide a complete repertoire of the secondary structures of all viroid species discovered to date.

## Results and discussion

The primary goal of this study was to generate the secondary structures of all known viroids species based on probing data at a single nucleotide resolution in solution. The adoption of a relatively fast protocol using fluorescently labelled oligonucleotides, and the capillary electrophoretic analysis of the cDNA resulting from the modified RNA templates, made the goal achievable. To pursue the effort of elucidating viroid secondary structures, the *in vitro* SHAPE-based protocol was initially applied to the members of the family *Pospiviroidae* for whom the structures in solution are not available (see Table 1 for the probed sequences and for further information).

**Table 1:** Viroids characterized in this study.

Viroid	Accession number of the probed sequence	Length (nt)	Genus	Closest probed viroid (sequence identity %)	First report of this species
Iresine viroid -1 (IrVd-1)	X95734.1	370	*Pospiviroid*	TCDVd (69.0)	[16]
Pepper chat fruit viroid (PCFVd)	NC_011590.1	348	*Pospiviroid*	PSTVd (68.5)	[17]
Tomato planta macho viroid (TPMVd)	K00817.1	360	*Pospiviroid*	TCDVd (81.1)	[18]
Mexican papita viroid (MPVd)	KF683198.1	359	*Pospiviroid*	TCDVd (84.8)	[19]
Coconut tinangaja viroid (CTiVd)	M20731.1	254	*Cocadviroid*	CCCVd (72.4)	[20]
Hop latent viroid (HLVd)	NC_003611.1	256	*Cocadviroid*	CBCVd (61.0)	[21]
Citrus viroid-V (CVd-V)	NC_010165.1	294	*Apscaviroid*	ASSVd (69.0)	[22]
Apple dimple fruit viroid (ADFVd)	NC_003463.1	306	*Apscaviroid*	CVd-III (72.3)	[23]
Australian grapevine viroid (AGVd)	NC_003553.1	369	*Apscaviroid*	CBLVd (58.9)	[24]
Grapevine yellow speckled viroid-1 (GYSVd-1)	NC_001920.1	366	*Apscaviroid*	CBLVd (63.8)	[25]
Grapevine yellow speckled viroid-2 (GYSVd-2)	J04348.1	363	*Apscaviroid*	GYSVd-1 (79.6)	[26]
Grapevine latent viroid	NC_028131	328	Unassigned	CVd-OS (75.0)	[27]
Dahlia latent viroid	JX263426.1	342	Unassigned	PCFVd (60.4)	[28]

**Fig 1:**
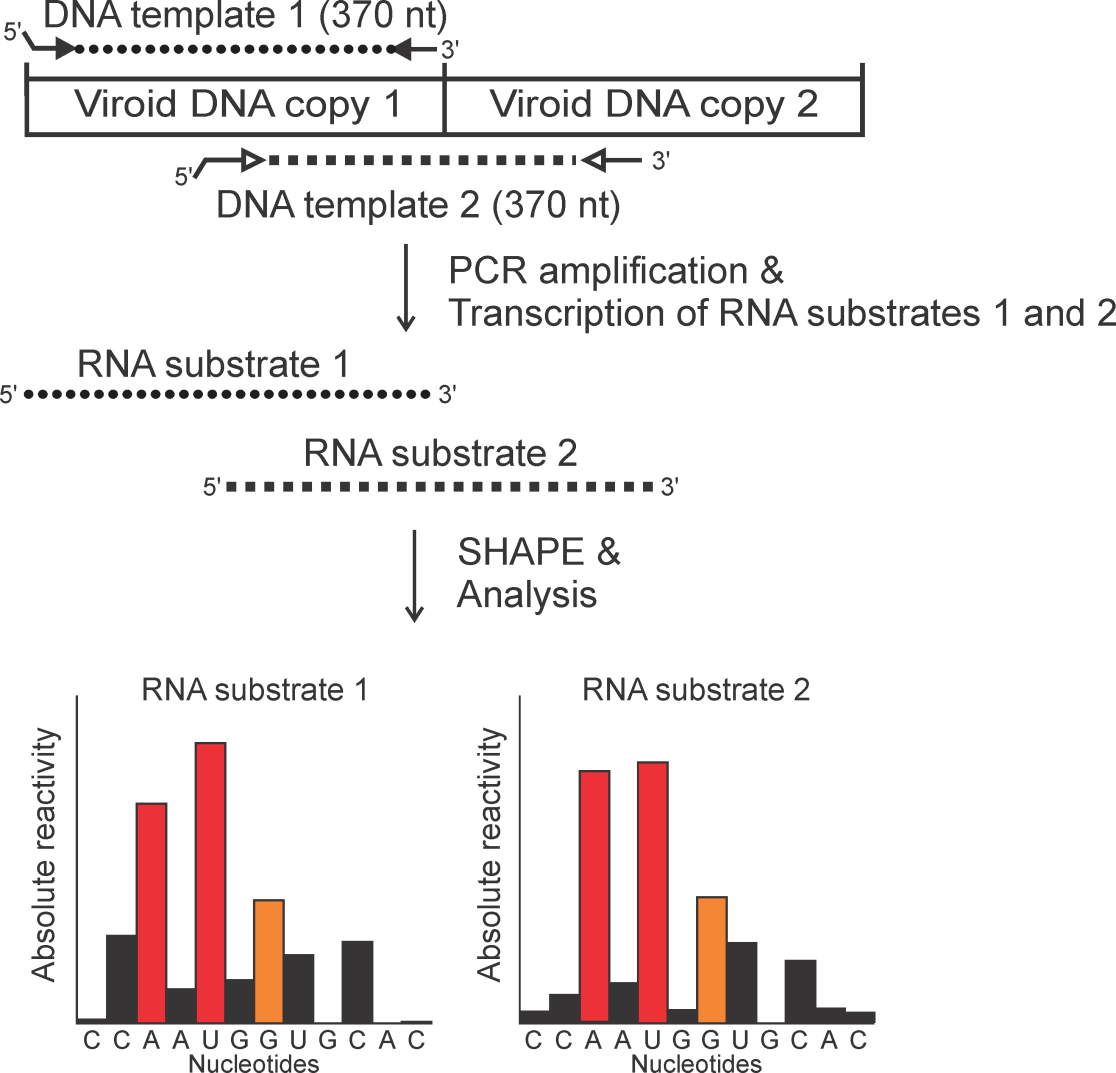
Schematic representation of a SHAPE probing experiment. The arrows show the primers used for the PCR amplification of the monomeric DNA templates 1 (full arrowheads) and 2 (white arrowheads). The RNA substrates were then produced by transcription from the T3 RNA polymerase promoter (represented by the raised extremity of the primers). The resulting RNA substrates 1 and 2 were then used in independent SHAPE reactions, and the reactivities of a sample of nucleotides for each RNA substrate are illustrated by the graphs. The black bars in the graphs represent nucleotides with low reactivities (0–0.40), the orange bars represent nucleotides with intermediate reactivities (0.40–0.85) and the red bars represent nucleotides with high reactivities (>0.85). Typical results for RNA species 1 and 2 were aligned on the original viroid sequence, and were then averaged to produce the final reactivity of each nucleotide and used in computer directed secondary structure prediction.

Briefly, full-length transcripts of (+) polarity were synthesized *in vitro* from a plasmid containing a head-to-tail dimeric copy of each viroid. Two different DNA templates were PCR amplified for every species (Fig 1). In each case, the forward primer included the T3 RNA polymerase promoter sequence at the 5’ end, and was always positioned so that the start site had at least one guanosine present so as to allow the subsequent transcription reaction. The purified transcripts were folded in solution in the presence of 10 mM MgCl_2_ at 37°C for 30 min so as to ensure complete folding. The experiments were also performed in the absence of magnesium with the goal of identifying any motifs that are affected by the presence of metal ions. Following the folding, the SHAPE reactions were performed using benzoyl cyanide (BzCN), as it reacts quickly and does not need to be deactivated because it is rapidly hydrolyzed [29]. Next, the primer extensions were performed using fluorescently labelled oligonucleotides. The resulting cDNA fragments were analyzed by capillary electrophoresis adjacent to a sequencing reaction. Since viroids are GC-rich, the sequencing reactions were performed in the presence of either ddGTP or ddCTP. As such, each primer extension reaction was analyzed twice, once with each ladder, thus facilitating the analysis and improving the alignment of the fluorescent peaks to the RNA sequence of the studied viroid. Moreover, primer extension reactions were also performed on transcripts in the absence of BzCN. These reactions served as negative controls, and permitted the subtraction of the background caused by premature termination by the reverse transcriptase.

The capillary electrophoresis data were analyzed using the QuSHAPE software [30]. The reactivity for each nucleotide was averaged from two different experiments for each primer used. Finally, the averaged reactivities were used as pseudo-energy constraints in the thermodynamic predictions, and the resulting most stable structures are presented in this report. The nucleotides with reactivities higher than 0.85 were considered as highly reactive, those with reactivity between 0.40 and 0.85 were considered as having an intermediate reactivity and those with values between 0 and 0.40 were considered as being unreactive. In order to confirm that the use of two distinct 5’ extremities did not affect the structure of the viroid, the reactivities of each nucleotide obtained with two transcripts with the differing 5’ termini were compared. Nucleotides with a low reactivity in one transcript and a high reactivity in the other were considered as having an inconsistent reactivity. Based on this, a percentage of reactivity consistency, that is to say the percentage of nucleotides with similar reactivities, regardless of the 5’ extremity of the transcript used, was determined. A level of 95% of reactivity consistency was considered as being satisfactory.

### Pospiviroid

**Fig 2:**
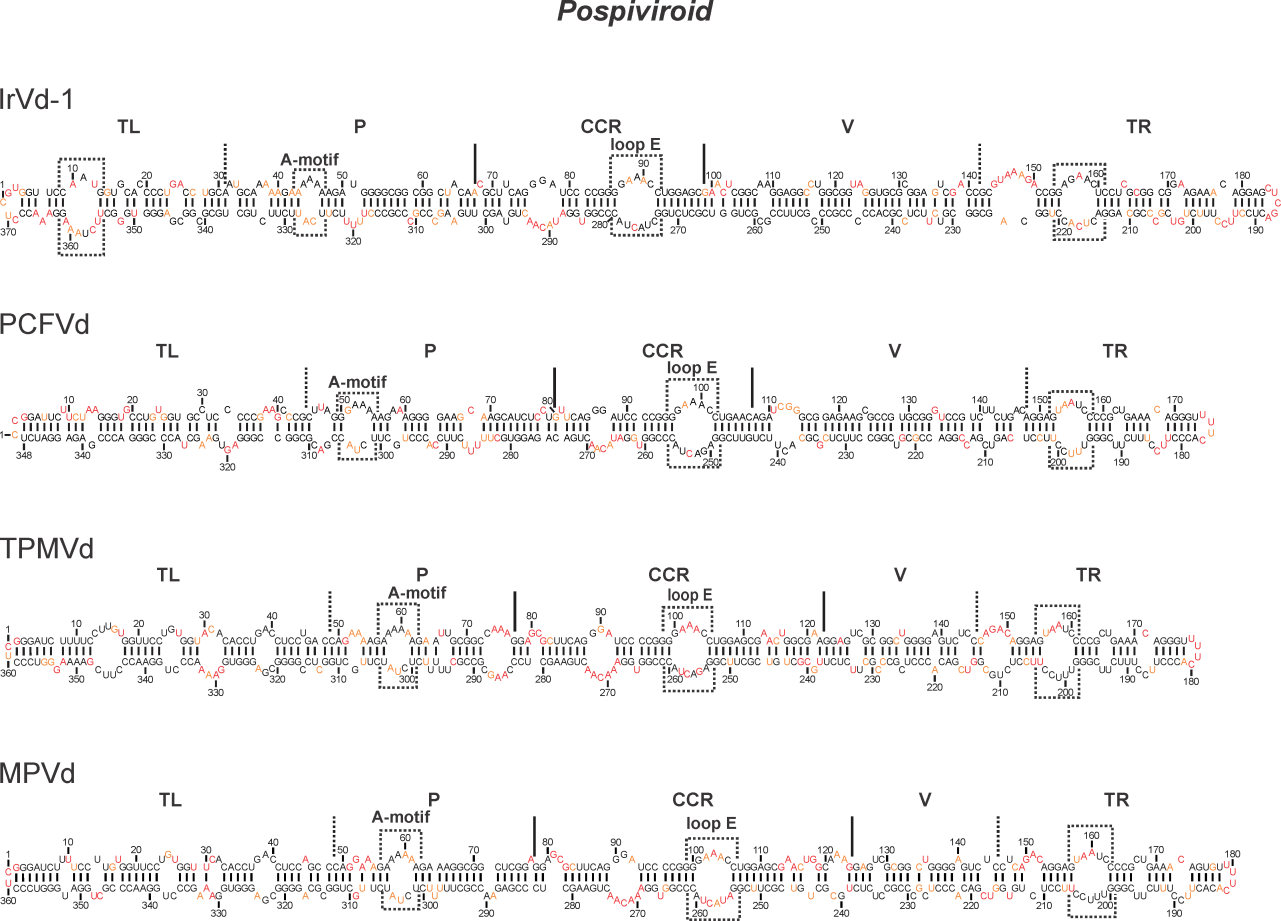
The elucidated secondary structures of 4 viroids from the genus *Pospiviroid*. The color of the nucleotide represents the level of accessibility as determined by SHAPE: namely the black nucleotides are of low reactivity (0–0.40), the orange nucleotides are of intermediate reactivity (0.40–0.85) and those in red are of high reactivity (>0.85). The different regions are marked by either full lines or dashed lines depending on whether they were previously published or were determined in this report, respectively. The boxed sections are the motifs referred to in the text.

Members of the genus *Pospiviroid* have been the most studied over the years. The type species of the family *Pospiviroidae*, and the first one discovered, is PSTVd. The structure of a sequence variant of PSTVd retrieved from dahlia plants and which causes mild symptoms in tomato plants, as well as that of a variant which causes intermediate symptoms in tomato plants, had previously been probed using the SHAPE technique[5,14]. In addition, the secondary structures of a sequence variant of the *Citrus exocortis viroid* (CEVd), of the *Tomato apical stunt viroid* (TASVd), of the *Chrysanthemum stunt viroid* (CSVd), of the *Columnea latent viroid* (CLVd) and of the *Tomato chlorotic dwarf viroid* (TCDVd) had been also reported based on SHAPE probing [14]. In the present work, the secondary structures of a sequence variant of the *Iresine viroid 1* (IrVd), the of *Pepper chat fruit viroid* (PCFVd), of the *Mexican papita viroid* (MPVd) and of the *Tomato planta macho viroid* (TPMVd) were elucidated in solution (Fig 2).

The *Iresine viroid 1* (IrVd) is composed of 370 nt (Table 1). It was found in symptomless *Iresine herbstii* [16]. This is the largest viroid of the *Pospiviroid*. The SHAPE data of the IrVd-derived transcripts with the two distinct 5’ extremities showed 98.1% of reactivity consistency, which is an excellent level. The resulting predicted structure showed that 11% of the nucleotides are in different structures as compared to the structure predicted without the SHAPE data (i.e. a single-stranded nucleotide that is instead in a double-stranded region or *vice versa*). The predicted secondary structure based on the SHAPE data is rod-like (Fig 2), as were seven of the other members of this genus that were solved. The one exception was CLVd, which had a branched TL region [14]. The presence of the CCR and the loop-E are characteristic of this genus. IrVd, however, shows some distinctive characteristics. Firstly, it has a large loop located at the beginning of the TL region that contains nucleotides 10–12 from the upper strand and 356–361 from the lower strand, which is unusual for viroids of this genus. Secondly, the A-motif of the P region is smaller than what is usually found in the *Pospiviroid*. Thirdly, it harbours a large loop (positions 155–159 and 215–219) that is inserted into the TR domain (see the boxed regions in Fig 2).

PCFVd is one of the recently discovered viroids that naturally infects bell pepper plants [17]. PCFVd infections in potato can give rise to small elongated and distorted tubers, while it causes the necrosis and the stunting of tomato plants [17]. The PCFVd probed transcripts showed 97% of reactivity consistency. The SHAPE-based prediction revealed a rod-like structure with a 7% difference as compared to that obtained without the probing data (Fig 2). The CCR that is characteristic of the genus *Pospiviroid* is present, with an asymmetrical loop (positions 86–87 and 250–255), and an isosteric variant of loop E (positions 97–101 and 250–255) is also present, as was previously reported [31]. The structure shows a typical A-motif located in the P region (positions 51–54 and 302–304). Interestingly, this structure comprises a highly based paired TL region, which is unique among all *Pospiviroid*. Although the TR region is like that of the other members of the genus *Pospiviroid*, it possesses a larger loop (positions 153–157 and 196–200), mostly due to the presence of additional uridines in the lower strand.

TPMVd is a species that shows 81% sequence identity with TCDVd (Table 1). Its distinct start sites affected the reactivity at only a few positions (95.8% reactivity consistency), and the folding obtained using the probing data gave a structure 12.5% different from that obtained without it. Overall, this viroid has an A-motif (positions 58–61 and 301–303) and a CCR (including a loop E at positions 100–104 and 254–259) that are similar to those of the two members of the *Pospiviroid* described above (Fig 2). It also possesses a large loop located in the TR domain (positions 156–160 and 199-203). The rest of the TR region is like that of almost all members of the *Pospiviroid* probed to date, with the exception of CLVd that has a branched structure.

The last viroid probed of this genus was MPVd, which was first found in the wild plants of *Solanum cardiophyllum* Lindl. in Mexico [19]. Although, MPVd and TPMVd share a high sequence identity, they were first proposed to belong to different species. This was based on the differences in the symptoms generated by both viroids in *Nicotiana glutinosa*, mainly the absence of the flower-breaking symptom in TPMVd infected plants, and on the inability of MPVd to replicate in *Gomphrena globosa* [19]. Recently, the flower-break symptoms, which are caused by many viroids in *N*. *glutinosa*, were found to be the same for both of these viroids. However, neither could infect *G*. *globosa*. Hence, it was thus proposed that both viroids belong to the same species [32]. The probings with two different transcripts bearing different 5’ extremities gave almost identical results with a reactivity consistency of 96.4%. The final structure presented in Fig 2 had a 16.7% difference with that predicted without SHAPE. The variant of MPVd selected for probing here has a 93% sequence identity with the TPMVd variant presented previously (Table 1). The resulting structure has all of the characteristics of a member of genus *Pospiviroid*, namely the A-motif (positions 58–61 and 302–304), the loop E (positions 101–105 and 255–260) and the TR region. The structures of MPVd and TPMVd are very much alike, even in the V region. However, differences were observed in the TL and P domains. Specifically, TPMVd possesses considerably larger loops at positions 13–17 and 344–347, 30–32 and 329–332 of the TL domain and at 73–76 and 284–288 of the P domain, which are absent in MPVd.

### Cocadviroid

**Fig 3:**
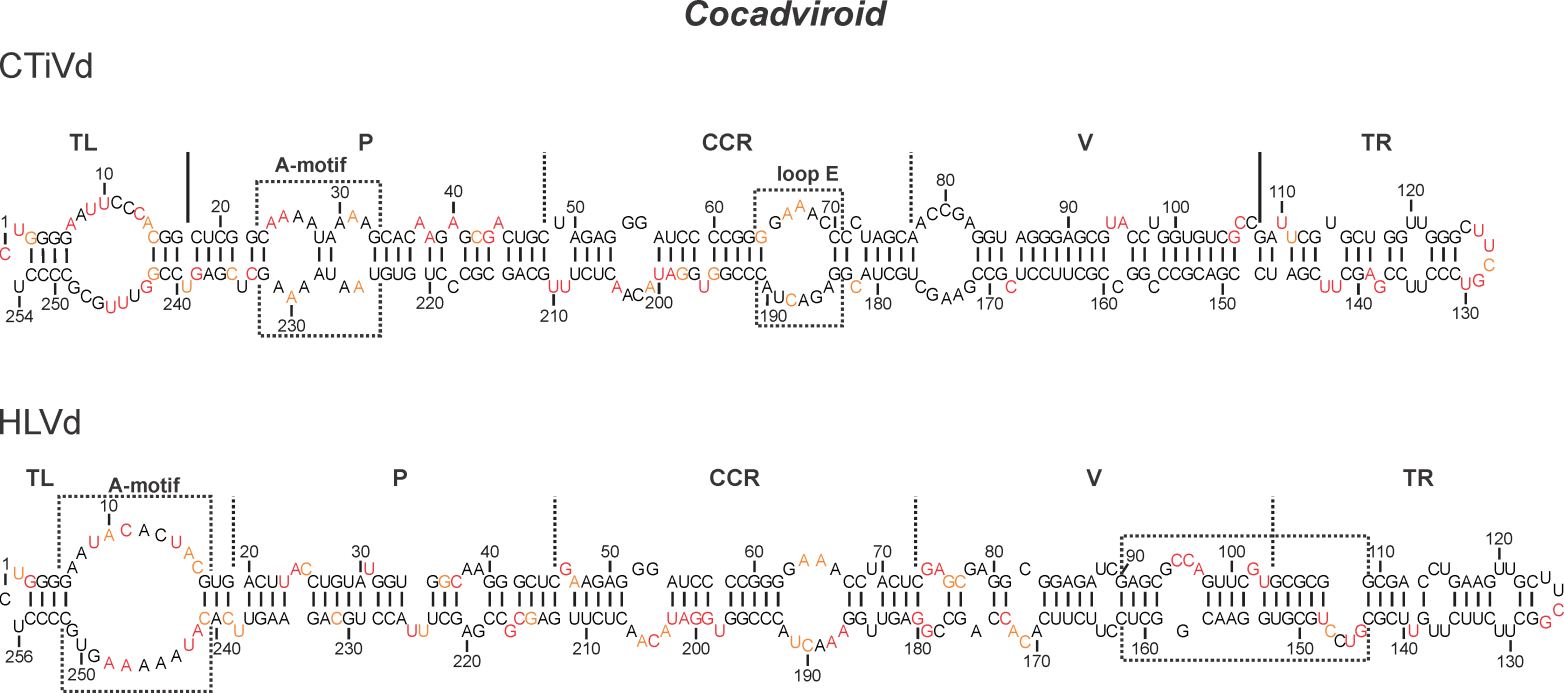
The determined secondary structures of viroids from the genus *Cocadviroid*. The color of the nucleotides represents the level of accessibility as determined by SHAPE: namely the black nucleotides are of low reactivity (0–0.40), the orange nucleotides are of intermediate reactivity (0.40–0.85) and those in red are of high reactivity (>0.85). The different regions are marked by either full lines or dashed lines depending on whether they were previously published or were determined in this report, respectively. The boxed sections are the motifs referred to in the text.

Prior to this study, the *Coconut cadang cadang viroid* (CCCVd) and the *Citrus bark cranking viroid* (CBCVd, previously known as the *Citrus viroid IV* (CVd-IV)) were the only two viroids of the genus *Cocadviroid* for which the secondary structure had been elucidated by SHAPE [13,14]. Conversely, the structures of both the *Coconut tinangaja viroid* (CTiVd) and the *Hop latent viroid* (HLVd) had not been probed previously. CTiVd and CCCVd have similar host ranges, but were discovered on different Pacific Islands [20]. They share 72% sequence identity, and cause different symptoms in their hosts. More precisely, CTiVd is responsible for the appearance of mummified nuts without kernels, while the CCCVd symptoms are mostly characterized by smaller, rounder scarified nuts. The two starting sites used for the probing had a very low impact on the folding of the RNA (i.e. 98.4% reactivity consistency). The probed structure of CTiVd is rod-like and has only a 4% difference with that predicted without the use of the SHAPE data (Fig 3). Nonetheless, the differences shown by the SHAPE probing have an impact on both the TL and the P domains. Specifically, there is the presence of a large loop from nucleotides 7 to 14 and 243 to 249 in the TL region of CTiVd that is absent in CCCVd. The CCCVd structure included a large A-motif located within the P region, which was replaced by two smaller A-motifs in the CTiVd SHAPE-based structure. Moreover, the CCR of CTiVd is similar to that of CBCVd with its isosteric loop E. Finally, the TR appears to be more base-paired than the rest of the viroid.

HLVd was first reported by Puchta *et al*. and was described as being a symptomless viroid in hops [21]. However, it was later shown that the HLVd infection of hops affected the production of secondary metabolites, such as alpha-bitter acid, as well as causing the production of smaller cones [33]. The two transcripts probed for HLVd provided almost identical data (i.e. 96% reactivity consistency). The differences between the structures predicted without and with the SHAPE data (Fig 3) were limited to only 7% of the nucleotides. Accordingly, HLVd folded into a rod-like structure. The structure of its CCR is characteristic of the *Cocadviroid*. The loop E is not isosterically viable because of the absence of a cytidine ending the upper loop [31]. Moreover, the presence of an unusually large A-motif located in the TL region (positions 7–16 and 242–251) was observed. The reactivities of the nucleotides in the A-motif of HLVd are very similar to those of the large loop in the TL domain of CTiVd, even though the sequences differ, suggesting possible non-canonical interactions. These would need to be confirmed with further experimentation such as three-dimensional structure determination. The right portion of the V domain and the left portion of the TR domain (positions 90–109 and 143–162) adopted the same structures as those previously reported for CbVd-2 [14]. According to the data presented here, all of the probed *Cocadviroid* fold into a rod-like structure that is characterized by the terminal left hairpin, the CCR structure, a smaller size and includes at least one large loop.

### Apscaviroid

Previous SHAPE probing data of some of the *Apscaviroid* members revealed diverse structures that are not simply rod-like. For example, the *Apple scar skin viroid* (ASSVd), which is the type species [25,34], the *Citrus dwarfing viroid* (CDVd) and the *Citrus viroid OS* (CVd-OS) have in solution structures that include a 3-way junction located in the TL domain [13,14]. Moreover, CVd-OS had a second 3-way junction located in the TR region. Conversely, both the *Pear blister canker viroid* (PBCVd) and the *Citrus bent leaf viroid* (CBLVd) appeared to fold into a classical rod-like structure that included a large loop in the TR domain [13,14]. In order to learn more about this peculiar genus, the SHAPE procedure was performed on five other *Apscaviroid* members, namely the *Citrus viroid V* (CVd-V), the *Apple dimple fruit viroid* (ADFVd), the *Australian grapevine viroid* (AGVd), the *Grapevine yellow speckle viroid 1* (GYSVd-1) and the *Grapevine yellow speckle viroid 2* (GYSVd-2) (see Table 1).

**Fig 4:**
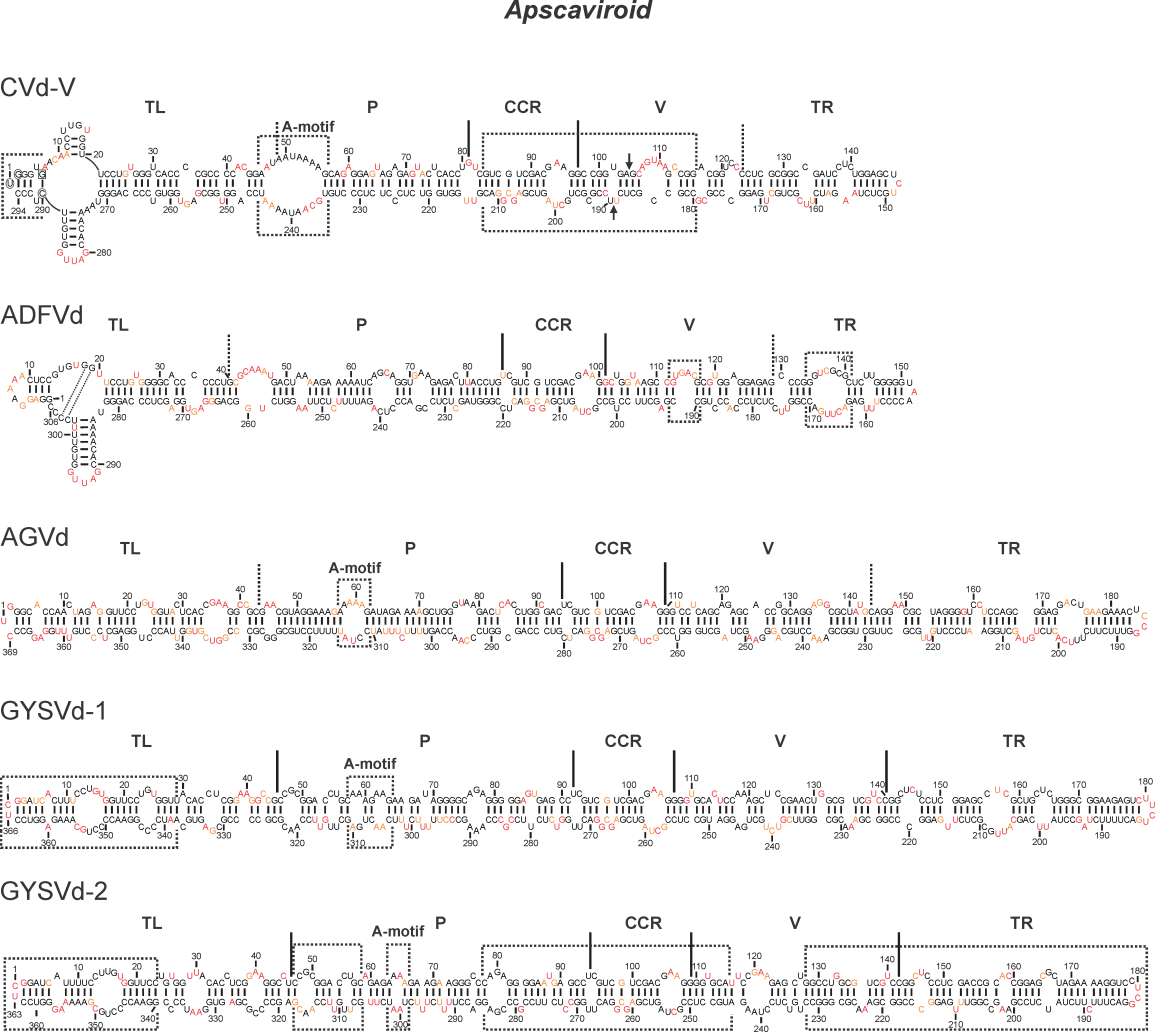
The determined secondary structures of viroids from the genus *Apscaviroid*. The color of the nucleotides represents the level of accessibility as determined by SHAPE: namely the black nucleotides are of low reactivity (0–0.40), the orange nucleotides are of intermediate reactivity (0.40–0.85) and those in red are of high reactivity (>0.85). The different regions are marked either by full lines or dashed lines depending on whether they were previously published or determined in this report, respectively. The boxed sections are the motifs referred to in the text. The circled nucleotides in the TL region of CVd-V mark the insertions as compared to ASSVd, and the boxed nucleotide represents a nucleotide variation. The arrowheads represent the position of the deleted block of nucleotides when compared to ASSVd. The possible interactions in ADFVd are represented by the dashed lines.

CVd-V, which is a 294 nt long RNA molecule (Table 1), is the only citrus infecting viroid that can infect *Atalantia citroides*, a plant that is usually resistant to viroid infection [22]. The probing of this viroid with two different 5’ extremities had low impact on the nucleotide reactivities as shown by the 97.6% reactivity consistency. While the structure predicted in the absence of SHAPE probing data was rod-like [22], that obtained when considering the SHAPE data included a 4-way junction motif located in the terminal left domain (Fig 4, see the cruciform). Overall, the difference between the structure deduced without SHAPE and that obtained with SHAPE was 15.3%. Its closest relative is ASSVd, with which it has 69% sequence identity (Table 1). The structure of this latter viroid was also branched, but with a 3-way junction [14]. The presence of an additional short hairpin in CVd-V is explained by the presence of a few extra nucleotides in the extremity of the TL domain (see the circled nucleotides in TL domain, Fig 4) that are absent in ASSVd. Additionally, the formation of this short hairpin is favoured by the base pairing of G_6_ with C_290_ (see the boxed nucleotide in TL domain, Fig 4). From left to right there are also several features that are specific to the CVd-V secondary structure. There is an unusually large A-motif that is caused by the adenosine rich regions located in both the upper and lower strands of the P domain (positions 47–55 and 236–244). This large A-motif was not very reactive in the presence of magnesium, but was in its absence, indicating a possible change of conformation. While both the CCRs and the V regions of CVd-V and ASSVd are very similar in terms of both sequence and structure, CVd-V is missing a block of 10 nt on both the upper and lower strands (indicated by the arrowheads in Fig 4). That said, this loss does not affect the conservation of the structures between CVd-V and ASSVd. Although nucleotide sequence differences were observed in the TR of CVd-V and ASSVd, the secondary structures looked alike.

ADFVd is a 306 nt long viroid responsible for the apple dimple disease in apple trees (Table 1) [23]. Overall, the structures of ADFVd differed by 15% when that predicted using the SHAPE data was compared to that predicted without it, and they were characterized by a 96.4% level of reactivity consistency when the two transcripts were compared. The resulting secondary structure is mostly rod-like, with the exception of a 3-way junction located in the TL region (Fig 4). The lack of reactivity of the single-stranded nucleotides located at positions 14 to 20 and 303 to 306 may support the formation of additional Watson-Crick base pairs between G_18_G_19_ and C_304_C_303_, as is illustrated by the dashed lines in Fig 4. This lack of reactivity was observed only in the presence of MgCl_2_. The sequence and structure of the TL domain appear to be similar to that of the CVd-OS variant obtained previously [14]. The P region of ADFVd included a relatively reactive asymmetrical loop that is formed by nucleotides 42 to 47 and 259 to 260. The CCR of ADFVd, which is formed by nucleotides 86 to 100 and 203 to 221, was characteristic of all members of the genus *Apscaviroid*. The V region includes an unusually reactive bulge that is formed by the four nucleotides located in positions 113 to 116 and is comparable to what is seen in CVd-OS [14]. Finally, the TR region exhibited a relatively large loop that is formed by nucleotides 135 to 140 and 163 to 168.

As opposed to the two viroids characterized above, AGVd, GYSVd-1 and GYSVd-2 folded into rod-like structures with several characteristic structural features (Fig 4). AGVd is an asymptomatic viroid associated with grapevines. Its probing had almost no inconsistent reactivities, as is demonstrated by a reactivity consistency of 97.8%. The SHAPE experiment yielded a structure with 7.3% of different nucleotide pairings as compared to that predicted in the absence of the chemical probing. The structure of the CCR (positions 95–110 and 263–279) of AGVd is characteristic of all other members of the genus *Apscaviroid*. However, the AGVd structure includes an A-motif located in the P domain (positions 58–61 and 312–314) that is identical to that of CVd-OS and is found in most of the members of the genus *Pospiviroid* [13,14].

Next, the GYSVd-1 type 3 sequence variant, which is often associated with yellow speckles disease in grapevines, was probed (Fig 4) [35]. This viroid is present in most grapevines and causes tiny chlorotic spots on the leaves that usually appear either at the end of the summer or during hot weather [36]. While both transcripts provided almost identical structures (reactivity consistency of 98.1%), the SHAPE probing yielded a structure that was significantly different from that obtained in the absence of probing data (18.9% difference). The high level of sequence identity and the similar reactivities of the nucleotides between the TL domains of GYSVd-1 and TPMVd caused both of these two domains to adopt a similar structure (see positions 1–29 and 338–366 of GYSVd-1). The CCR structure was similar to that of the other members of the genus *Apscaviroid* (positions 92–107 and 255–273). In fact, the characteristic feature of the GYSVd-1 structure was a P region that included two small loops of two nucleotides each, reminiscent of an A-motif, located on both stands and separated by a small, double-stranded helix (positions 58–68 and 299–310).

GYSVd-2 was the last member of this genus to be probed (Fig 4). The resulting RNA probings of the two transcripts had 96.7% reactivity consistency, and there was an 11.8% difference between the structure predicted without SHAPE and that with it. The variant selected here has 79.6% sequence identity with GYSVd-1 (Table 1) [26]. Some parts of their structures were similar, for example the TL domain located at positions 1–23 and 340–363 and which is also similar to the TL domain of TPMVd (Fig 2). Also, the left portion of the P domain of GYSVd-2 is identical to that of GYSVd-1 in terms of both sequence and structure (positions 47–58 and 306–318). However, only a small A-motif was found to be present in GYSVd-2 (positions 64–65 and 300–301). The right portions of the P region, right up to the CCR, of these two viroids from (positions 78–94 and 268–285) showed more differences at the sequence level than at the structural level. The CCR is identical to that of those of the other *Apscaviroid*, and the left portion of the V region (positions 109–115 and 225–249) is identical to that of GYSVd-1. Moreover, the structures of the right half of the V domain and of the TR domain located between positions 128 to 232 of GYSVd-2 were very similar to those of GYSVd-1, with the exception of the bulge and loop seen in GYSVd-2 (positions 158–160 and 201–204) and the loop seen in GYSVd-1 (positions 156–158 and 205–208).

In brief, all of the members of the genus *Apscaviroid* possess similar CCRs. Their TL regions can be either branched, as is observed with CVd-V and ADFVd, or rod-like as is seen with the grapevine infecting viroids AGVd, GYSVd-1 and GYSVd-2. Distinctive structural characteristics were also observed in their P domains. For example, CVd-V possesses a large A-motif, while AGVd possesses the same A-motif as the members of the genus *Pospiviroid* do. Both the V and TR regions are different for all viroids. Specifically, all viroids characterized in this study exhibited rod-like structures in these domains, but CVd-OS, which was probed previously, is branched [14].

### Structural hallmarks of each genus

**Fig 5:**
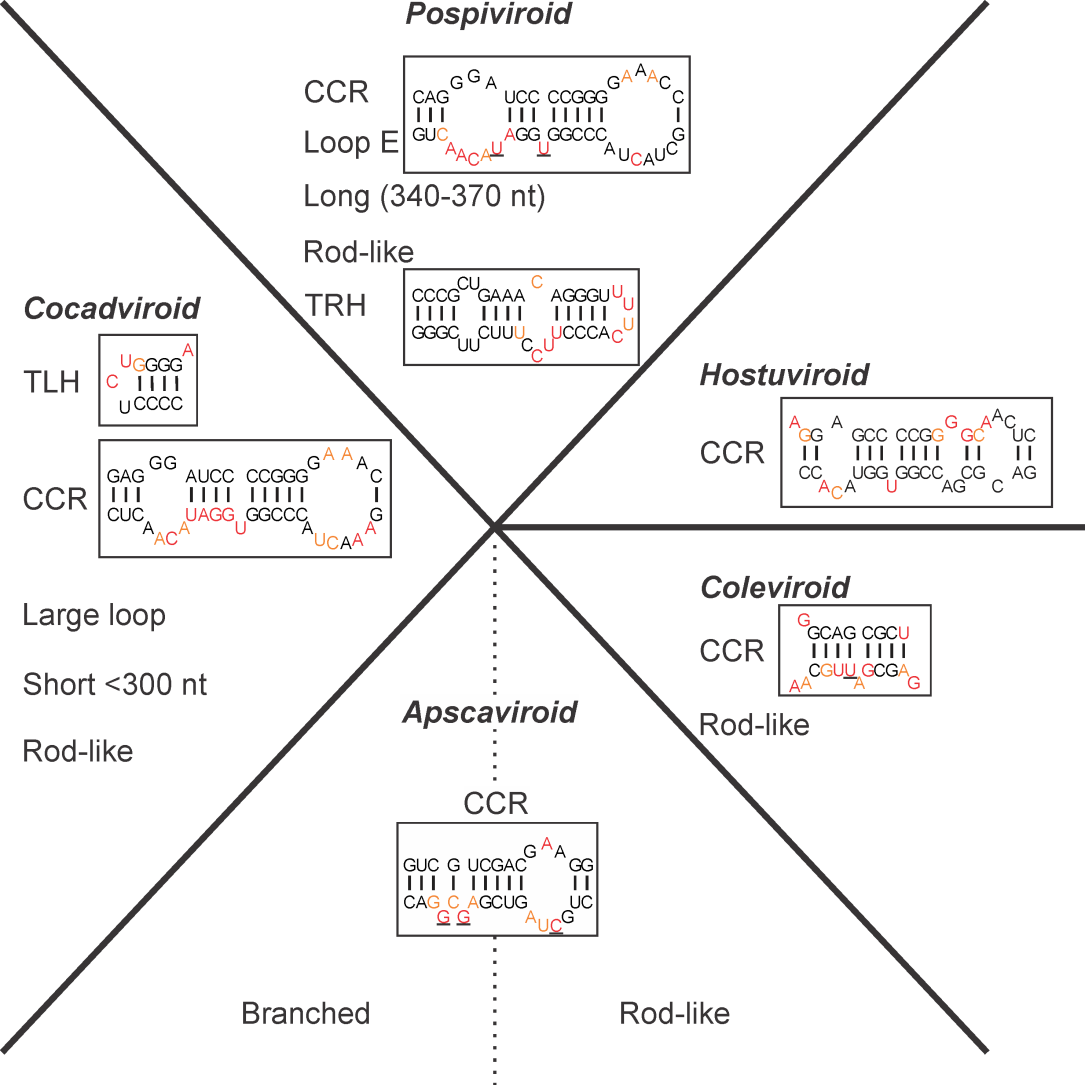
Classification of the *Pospiviroidae* members based on their structural hallmarks. The boxed structures are representative examples for each genus. The color of the nucleotide represents the level of accessibility as determined by SHAPE: namely the black nucleotides are of low reactivity (0–0.40), the orange nucleotides are of intermediate reactivity (0.40–0.85) and those in red are of high reactivity (>0.85). The underlined nucleotides are very reactive (>2.0). Structure of the CCR of the *Coleviroid* is presented as in previous report [14].

After studying the structures in solution of most *Pospiviroidae*, we attempted to identify any structural hallmarks that could be useful for the classification of viroids into the different genera (Fig 5). Clearly, the CCR is the most important structural hallmark for the differentiation of the different genera. In fact, it is well known that the CCR of viroids is composed of a well-conserved sequence in both the upper and lower strands of the viroid [2]. Also, the structure of this motif, as based on the SHAPE data, is highly conserved and the key positions are always very highly reactive (>2.0). A possible explanation for this is that the formation of a three-dimensional conformation that is highly favourable for 2’hydroxyl acylation [37]. However, there are also other structural hallmarks that can be used in order to help group a viroid into a genus. Briefly, all of the *Pospiviroid* have similar CCRs that include a loop E, a TR hairpin (TRH) and are relatively long in terms of size. The CCRs of the *Cocadviroid* are very similar to those of *Pospiviroid*, and the members of this genus are shorter and contain at least one large loop and a common TL hairpin. Both the *Coleviroid* and *Hostuviroid* have their own distinct CCRs. All of the members of the genus *Apscaviroid* have similar CCRs and belong to either the rod-like group or the TL branched group. There is also the terminal conserved region (TCR) which is composed of a sequence that is more or less conserved and that can be found in the TL region of all viroids of greater than 300 nt in size, but since it is not found in the lower strand it is not associated with a structural motif and was not used here to classify species.

As reported previously, the classification of CLVd can be challenging [2,38]. This species was originally proposed as a *Pospiviroid*. However, in a previous probing study, the CLVd structure did not possess a loop E and its TRH was not reminiscent of those of the other *Pospiviroid* as it was branched [14]. Considering that CLVd harbours the same CCR as HSVd, the only member of the *Hostuviroid*, the suggestion here is that it should be classified has an *Hostuviroid*, as has been proposed previously [14].

### Classification of two novel viroids

**Fig 6:**
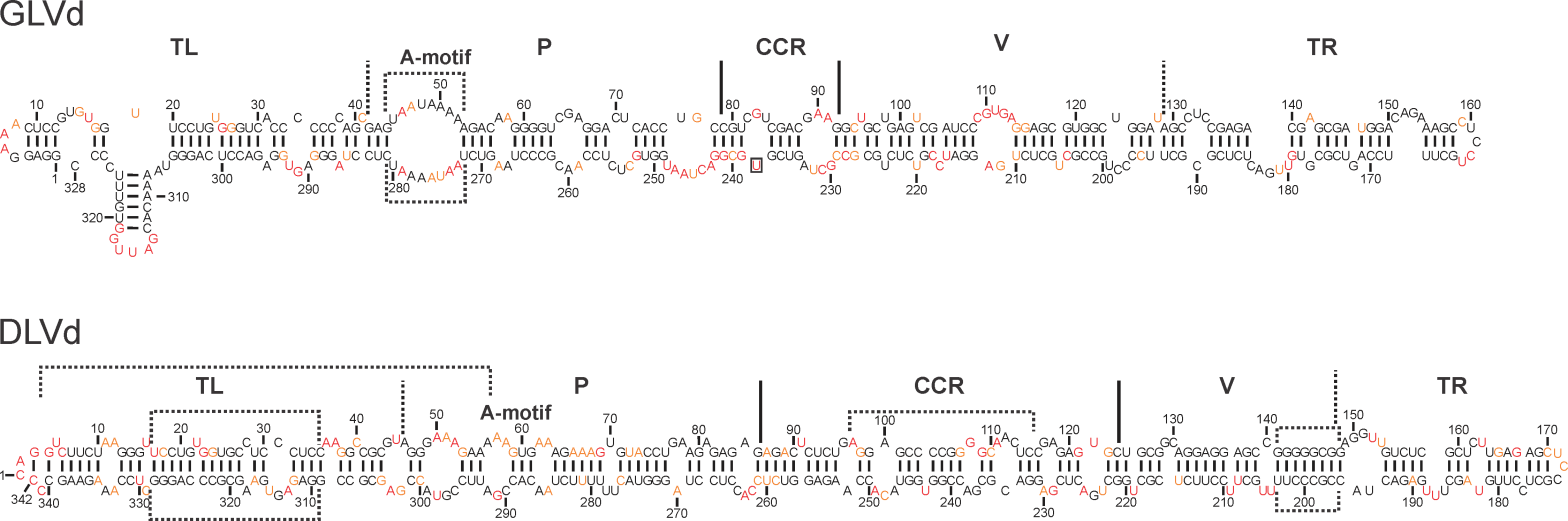
The determined secondary structures of two novel viroids. The most stable structures of GLVd and DLVd as elucidated by SHAPE probing. The color of the nucleotides represents the level of accessibility as determined by SHAPE: namely the black nucleotides are of low reactivity (0–0.40), the orange nucleotides are of intermediate reactivity (0.40–0.85) and those in red are of high reactivity (>0.85). The different regions are marked by either full lines or dashed lines depending on whether they were previously published or were determined in this report, respectively. The boxed sections are the motifs referred to in the text.

The *Grapevine latent viroid* (GLVd), an asymptomatic species of 328 nt, was recently reported by Zhang *et al*. (Table 1) [27]. The two transcripts possessing different 5’ extremities used for the probing revealed an accuracy of 98%. The percentage of change between the structures predicted with and without SHAPE is 11% (Fig 6). The probed structure revealed a branched TL domain very similar to those of CVd-OS [14] and ADFVd (described in this report, Fig 4). The P domain possesses a large motif-A like that seen in CVd-V (Fig 4). Interestingly, the CCR of GLVd was virtually identical to that of *Apscaviroid*, with one modification, specifically that there was a uridine at position 242 instead of an adenosine. The CCR of *Apscaviroid* is composed of two bulges of one guanine each followed by a stem of five base pairs and a loop (Fig 5), while that of GLVd is composed of a loop followed by a stem of four nucleotides and the loop (Fig 6). Therefore, the suggestion here in to propose that GLVd be classified as a member of the genus *Apscaviroid* because of its structure and without considering any other characteristic such as its sequence. It would be interesting to change the uridine located at position 242 to an adenosine in order to render it a more traditional CCR and to see the impact of this change on the viroid’s biological cycle as well as the effect on its host.

Another recently discovered viroid is the *Dahlia latent viroid* (DLVd) [28]. This viroid is composed of 342 nt and was discovered in asymptomatic dahlia plants (Table 1). The infection of other *Pospiviroid* hosts, such as tomato and cucumber, indicated that DLVd could only replicate in the dahlia plant [28]. The sequence identity of this viroid is low, being only 60.4% identical to its closest relative, PCFVd, and 57% with HSVd. Comparison of the sequences indicated that the upper strand of the TL domain was conserved between DLVd and PCFVd (nucleotides 4–59), and that the upper and lower strands of the CCR were conserved between DLVd and HSVd. Since PCFVd belongs to the *Pospiviroid* genus and HSVd belongs to the *Hostuviroid* genus, the classification of DLVd into the right genus is not obvious. Therefore, it was decided to probe this viroid in order to identify its structural hallmarks. The probing of full-length transcripts with two different 5’ extremities gave a reactivity consistency of 96%. The structure predicted using the SHAPE data is rod-like with 17.8% of change from the structure predicted without SHAPE (Fig 6). The probing of DLVd revealed the presence of a TL region and an A-motif in the P region similar to those of PCFVd (Fig 2) even though there is less sequence conservation of the lower strand. The CCR is comparable to those of HSVd and CLVd. Moreover, DLVd does not possess a potential loop E. The V domain of DLVd has a G-rich region, like that of CLVd, from positions 141 to 148. The TRH present in all viroids of the genus *Pospiviroid* is not present in DLVd. Since DLVd does not possess the TRH, but has a CCR like CLVd and HSVd, it appears reasonable to classify it into the *Hostuviroid*.

### Concluding observations

The secondary structures, as predicted based on SHAPE data, of thirteen viroid species for which no in-solution mapping data was available are reported here. Clearly, the procedure is now well-established and optimized. For viroids in solution, the advantage of using two transcripts with different 5’ extremities is that it produces data on each nucleotide. The average of reactivity consistency of 97.1% clearly demonstrates that, for these transcripts, folding in solution was not affected by starting site. Also, it shows that viroids tend to fold into the same structure regardless of starting site. However, to achieve a high level of reactivity consistency, starting sites must be selected carefully. First, the 5’ extremity needs to begin with at least one guanine for transcription. Second, it must be in a stable region such as a stem or the loop of an hairpin. Finally, the two starting sites must be far apart to obtain reactivity data for each nucleotide.

Together with previous data reported in three distinct studies [9,13,14], this report provides a complete compendium of the secondary structures predicted using SHAPE data for one representative sequence variant for all viroids reported to date in the literature. For all probed viroids, a percentage of change between predictions without and with SHAPE was observed. For this report, the average percentage of change is established at 12.0%. This value is lower than previously reported for members of the *Avsunviroidae* (23%) and of the *Popiviroidae* (20%) families [9,14]. This is mostly due to the nature of the probed viroid species. Firstly, *Avsunviroidae* are typically branched viroids with secondary structures that tend to be more challenging to predict accurately. Secondly, the species of the *Pospiviroidae* family that were probed previously [14], were selected for probing because of their peculiar predicted secondary structures. Overall, prediction with SHAPE data differed by 18% from prediction without SHAPE data, when considering the three reports [9,14] and this one. This clearly underlines the importance of acquiring in solution probing data to support the secondary structure of viroids. It was possible to deduce the structural hallmarks of each viroid genus by considering the structures of all viroids species (Fig 5). On the basis of the structural hallmarks, it was suggested that GLVd belongs to the *Apscaviroid*, while DLVd belongs to the *Hostuviroid*. Since DLVd seems to replicate exclusively in dahlia, whereas HSVd is able to replicate and cause symptoms in a large range of hosts, the inclusion of CLVd in the *Hostuviroid* group should no longer be limited by its biological features (such as host range) or by this genus’ absence of the TCR or presence of TH. Importantly, whenever a new viroid is reported, a relevant predicted secondary structure based on SHAPE probing can also be reported, and determination of the structural hallmarks should add confidence in its proper classification.

Finally, and very importantly, having a complete repertoire of the secondary structures of all viroids should be a great reference point for many biological studies, permitting an understanding of what is happening at the nucleotide level of a relevant structure. Eventually, these SHAPE data could be used to derive possible three-dimensional conformations for each viroid domain. Moreover, the probing of very similar viroids like TPMVd and MPVd provided structures that are similar. Therefore, someone working with a sequence variant highly similar to one of the variants reported in this compendium may consider using the SHAPE reactivity obtained for the latter to guide the prediction of that in his experiments in the regions of the viroid with high sequence identities. Guiding the structure prediction may be helpful in understanding some experimental results, but the best strategy remains to perform SHAPE probing in order to confirm the structure.

## Materials and methods

### Preparation of the transcription templates

For each viroid probed, a plasmid containing a head-to-tail dimer of the desired viroid was constructed by gene synthesis (GeneArt, Life Technologies or Bio Basics). In order to produce viroids with different start sites, an amplification of the DNA was performed by PCR. The primers were carefully selected so as to encompass the entire viroid, to have the start sites be a guanine residue (to ensure the success of the subsequent transcription reaction) and to be located in a region where the effect of the start site on the overall structure would be minimal. The primers were usually designed so as to position the 5’ end of the transcript near either the right (TR) or the left terminal loops (TL) of the viroid's rod-like structure. The forward primers contained the T3 RNA polymerase promoter sequence at their 5’ ends for use in the production of the RNA (see S1 Table for a detailed list of the primers used). To amplify the viroids, plasmid DNA (5 ng) was added to purified *Pfu* DNA polymerase (2 μl) in a buffer containing 20 mM Tris-HCl pH 8.8, 10 mM (NH_4_)_2_SO_4_, 10 mM KCl, 0.1% Triton X-100, 20 mM dNTPs, 200 mM MgSO_4_ and 200 μM of each of the primers. The amplification was performed in a thermocycler using a program of 1 min at 95°C, 1 min at 65°C, 45 s at 72°C for 35 cycles, followed by a final elongation period of 5 min at 72°C. A fraction of the amplified product was used to verify the integrity and size of the amplified DNA by electrophoresis on a 1% agarose gel. The remaining fraction of the amplification reaction was ethanol precipitated, air dried and dissolved in nanopure water.

### Transcription of the RNA

The transcriptions of the amplified DNA templates were performed in the presence of “in-house” purified T3 RNA polymerase (2 μl, 1 μg/μl), pyrophosphatase (0,02 U, Roche Diagnostics) and RNAseOUT (40 U, Life Technologies) in transcription buffer containing 80 mM HEPES-KOH (pH 7.5), 24 mM MgCl_2_, 2 mM spermidine, 40 mM DTT and 5 mM of each NTP. The reactions were incubated at 37°C for 90 min. DNAse RQ1 (3 U, Promega) was then added, and the mixture incubated at 37°C for 15 min to degrade the DNA template. A phenol-chloroform extraction was then performed, followed by an ethanol precipitation of the RNA. The RNA was further purified by denaturing gel electrophoresis (5% acrylamide and 8 M urea). The RNA in the gel was visualized by UV-shadowing, the gel excised and the RNA eluted in elution buffer (500 mM NH_4_OAc, 10 mM EDTA and 0.1% sodium dodecyl sulphate (SDS)) overnight at room temperature. Lastly, the eluted RNA was precipitated with ethanol, dried and dissolved in 100 μl TE 0.5X (5 mM Tris-HCl pH 5.5 and 500 μM EDTA). The RNA concentration was determined by UV spectrophotometry at 260 nm using a Nanodrop spectrophotometer.

### SHAPE probing

The SHAPE reaction was performed using 5 pmol of RNA in 8 μl of TE 0.5X. The RNA was unfolded at 95°C for 3 min, and was then quickly put on ice for 5 min. After the addition of 1 μl of folding buffer (500 mM Tris-HCl pH 7.5, 500 mM NaCl) the samples were incubated at 37°C for 5 min in a pre-folding step. Next, 1 μl of 100 mM MgCl_2_ was added and the folding reaction was incubated at 37°C for 30 min. For the samples without magnesium, water was added instead of MgCl_2_. A fresh solution of the SHAPE reagent BzCN (600 mM in DMSO) was prepared, and 1 μl was added to the folded RNA. In the negative control, 1 μl of DMSO was added in place. The SHAPE reactions were completed in 1 s at 37°C and did not need to be deactivated. Glycogen (1 μl,) was added to the reactions and the RNA was ethanol precipitated. The RNA pellets were washed with 70% ethanol, air-dried and dissolved in 10 μl of TE 0.5X.

For the primer extension reaction, the RNA was heated at 95°C for 2 min and snap-cooled on ice for 5 min. The fluorescent primer (1 pmol) was hybridized to the RNA by incubating the mixture at 52°C for 5 min, then at 37°C for 5 min and finally at 4°C for 1 min. Following this step, the reagents required for the primer extension reaction were added: 4 μl of 5X first strand buffer (250 mM Tris-HCL pH 8.3, 375 mM KCl, 15 mM MgCl_2_), 1 μl of 10 mM dNTPs, 1 μl of 100 mM DTT and 2 μl of DMSO. For the preparation of the sequencing reactions, 5 pmol of RNA were diluted in 9 μl of TE 0.5X and 1 μl of either ddGTP (5 mM) or ddCTP (10 mM) was added. Both the primer extension and the sequencing reactions were incubated for 1 min at 52°C prior to the addition of 140 units of *SuperScript III* (Life Technologies). The primer extension reactions were performed at 52°C for 30 min, and were stopped by the addition of 1 μl of 2 M NaOH and incubation at 95°C for 5 min in order to degrade the RNA. Following this step, 80 μl of water and 1 μl of glycogen were added prior the ethanol precipitation. The DNA pellets were washed twice with 70% ethanol and air-dried. The electrophoresis of the cDNA was performed at a sequencing and genotyping facility (*Plateforme de séquençage et de génotypage*, *Centre de recherche* (CHUL) (Québec city)). The DNA pellets are dissolved in a mixture of 10 μl each of H_2_O and formamide with the addition of a Lyz labelled control DNA ladder (Life Technologies). Each SHAPE (+) and SHAPE (-) reactions was electrophoresed in the presence of one sequencing reaction on ABI 3100 Genetic Analyzer (Life Technologies). The electrophoresis was then repeated with the SHAPE (+) and SHAPE (-) reaction in the presence of the other sequencing reaction. The analysis of the electropherograms was performed”in house” using the default parameters of the QuSHAPE software [30].

The normalized reactivity of each nucleotide was averaged between the replicates (averaged data S1 File). For each transcript, the reactivity of each nucleotide was evaluated to determine the effect of the two start sites on the resulting structure. If a stretch of more than four nucleotides was found to be unreactive (<0.40) with one transcript, but highly reactive (>0.85) with the other, then the whole experiment was repeated using a third transcript possessing a different 5’ extremity. The reactivities obtained for each transcript were averaged, and were used as pseudo-energy constraints with the default slope (1.8 kcal/mol) and intercept (-0.6 kcal/mol) values in the Fold tool of the RNAstructure 5.6 software [39]. The structures with the lowest Gibbs free energies were used for analysis. Finally, the reactivities obtained without magnesium were compared to those obtained in its presence in order to evaluate whether or not the absence of magnesium caused changes in the structure.

## Supporting information

S1 Table List of the primers used in this study. F1, F2 and F3 are the forward primers. The number at the end of the primer name is the first nucleotide in 5’ of the transcript as numbered according to the circular viroid. F1, F2 and F3 primers contain the polymerase T3 promoter (see the underlined sequences). R1, R2 and R3 are the reverse primers. 5’ fluorescent primers (6-Fam or VIC) of the R1, R2 and R3 primers were used during the primer extension reactions.
(DOCX)

S1 File SHAPE data for each viroid of this study. Data are presented in a two-column format for each viroid, with position numbers in the first column, and corresponding reactivity value in the second column.
(XLSX)
