# Supplementary material for: Classification of the Pospiviroidae based on their structural hallmarks
Source: PLoS One. 2017 Aug 4;12(8):e0182536. doi: 10.1371/journal.pone.0182536 (PMC5544226; doi:10.1371/journal.pone.0182536)
Supplement: S1 Table — F1, F2 and F3 are the forward primers. The number at the end of the primer name is the first nucleotide in 5’ of the transcript as numbered according to the circular viroid. F1, F2 and F3 primers contain the polymerase T3 promoter (see the underlined sequences). R1, R2 and R3 are the reverse primers. 5’ fluorescent primers (6-Fam or VIC) of the R1, R2 and R3 primers were used during the primer extension reactions. (DOCX) [file pone.0182536.s001.docx]

**S1 Table. List of the primers used in this study**

| **Primer name** | **Sequence** |
| --- | --- |
| IrVd_F1_4 | 5’-AATTAACCCTCACTAAAGGTTCCAATGGTGCACCCCTG-3’ |
| IrVd_R1 | 5’-ACGAGGGTTTCCTTTAGAAGCCC-3’ |
| IrVd_F2_180 | 5’-AATTAACCCTCACTAAAGGAGCTCGACTCCTTCCTTTC-3’ |
| IrVd_R2 | 5’-TGTTTCTTCCGCCGCGAGGAG-3’ |
| PCFVd_F1_3 | 5’-AATTAACCCTCACTAAAGGATTCTTCTAAGGGTGCCTG-3’ |
| PCFVd_R1 | 5’-GGAGATCCTCTCGGGTCCC-3’ |
| PCFVd_F2_170 | 5’-AATTAACCCTCACTAAAGGGTTTTCACCCTTCCTTTC-3’ |
| PCFVd_R2 | 5’-TGTTTCAGCGGGGATTACTC-3’ |
| TPMVd_F1_173 | 5’-AATTAACCCTCACTAAAGGGTTTTCACCCTTCCTTTCTTC-3’ |
| TPMVd_R1 | 5’-TGTTTCAGCGGGGATTACTC-3’ |
| TPMVd_F2_98 | 5’-AATTAACCCTCACTAAAGGGAAACCTGGAGCGAACTGGC-3’ |
| TPMVd_R2 | 5’-CGGGGATCCCTGAAGCGCTC-3’ |
| MPVd_F1_175 | 5’-AATTAACCCTCACTAAAGTGTTTTCACACTTCCTTTCTTC-3’ |
| MPVd_R1 | 5’-TGTTTCAGCGGGGATTACTC-3’ |
| MPVd_F2_99 | 5’-AATTAACCCTCACTAAAGGGAAACCTGGAGCGAACTGGC-3’ |
| MPVd_R2 | 5’-CGGGGATCCCTGAAGCGCTC-3’ |
| CTiVd_F1_3 | 5’-AATTAACCCTCACTAAAGGGGAATTCCCACGGCTCGG-3’ |
| CTiVd_R1 | 5’-AGAGGGGCGCAAACCGGAC-3’ |
| CTiVd_F2_122 | 5’-AATTAACCCTCACTAAAGGGCTTCGTCCCTTCCGAGC-3’ |
| CTiVd_R2 | 5’-AACCAGCACGAATCGGCGA-3’ |
| HLVd_F1_3 | 5’-AATTAACCCTCACTAAAGGGGAATACACTACGTGACTTACC-3’ |
| HLVd_R1 | 5’-AGAGGGGCACTTTTTATGTG-3’ |
| HLVd_F2_127 | 5’-AATTAACCCTCACTAAAGGCTTCTTCTTGTTCGCGTCC-3’ |
| HLVd_R2 | 5’-GAAGCAACTTCAGGTCGCCG-3’ |
| CVd-V_F1_2 | 5’-AATTAACCCTCACTAAAGGGTGAACAACCTTGTGGTTC-3’ |
| CVd-V_R1 | 5’-AGGGAGAACACCAATCGTG-3’ |
| CVd-V_F2_142 | AATTAACCCTCACTAAAGGAGCTCTGCTCTAAGATC-3’ |
| CVd-V_R2 | 5’-AGAGGATCGGCCGCGAGGG-3’ |
| ADFVd_F1_1 | 5’-AATTAACCCTCACTAAAGGAGGAAAACTCCGTGTGG-3’ |
| ADFVd_R1 | 5’-GGGGAAAACACCAATCGTG-3’ |
| ADFVd_F2_146 | 5’-AATTAACCCTCACTAAAGGGGGTAACCCCTTTGAGAC-3’ |
| ADFVd_R2 | 5’-AAGAGCGCGACCCGGGCTC-3’ |
| AGVd_F1_2 | 5’-AATTAACCCTCACTAAAGGGCACCAACTAGAGGTTCC-3’ |
| AGVd_R1 | 5’-AGGGCCTCCAAACAGGGAG-3’ |
| AGVd_F2_187 | 5’-AATTAACCCTCACTAAAGGTTTCTTCTTTCACTCTGTAGC-3’ |
| AGVd_R2 | 5’-GGAGTTTCTTCAGTCCTCCG-3’ |
| GYSVd-1_F1_2 | 5’-AATTAACCCTCACTAAAGGATCACTTTCCTGTGGTTCC-3’ |
| GYSVd-1_R1 | 5’-GAGGACCTCTTTGCAGGGG-3’ |
| GYSVd-1_F2_167 | 5’-AATTAACCCTCACTAAAGGGCGGAAGAGTCTTCTGAC-3’ |
| GYSVd-1_R2 | 5’-AGAGCAGCGAGGCTCCGAGG-3’ |
| GYSVd-2_F1_2 | 5’-AATTAACCCTCACTAAAGGATCATTTTCCTTGTGGTTC-3’ |
| GYSVd-2_R1 | 5’-GAGGACCCTTTTTCGCAGGGG-3’ |
| GYSVd-2_F2_182 | 5’-AATTAACCCTCACTAAAGGACTTTCTTCTATCTCCGAA-3’ |
| GYSVd-2_R2 | 5’-GAGGACCTTTTCTAGCGCTCC-3’ |
| GLVd_F1_1 | 5’-AATTAACCCTCACTAAAGGAGGAAACTCCGTGTGGT-3’ |
| GLVd_R1 | 5’-GGGGAAACACCAATCGTGTT-3’ |
| GLVd_F2_213 | 5’-AATTAACCCTCACTAAAGGATCCGTCTCTGCGCCGCT-3’ |
| GLVd_R2 | 5’-TCAGAGCAGCGGCAGGGGAAG-3’ |
| DLVd_F1_3 | 5’-AATTAACCCTCACTAAAGGTCTTCTAAGGGTTCCTGTG-3’ |
| DLVd_R1 | 5’-TGGGGCTTCTTTGGAGCCCTG-3’ |
| DLVd_F2_231 | 5’-AATTAACCCTCACTAAAGGACGCGACCGGTGGTACACC-3’ |
| DLVd_R2 | 5’-TCGAGTCAGCCAGCGAGAAGG-3’ |

F1, F2 and F3 are the forward primers. The number at the end of the primer name is the first nucleotide in 5’ of the transcript as numbered according to the circular viroid.

F1, F2 and F3 primers contain the polymerase T3 promoter (see the underlined sequences).

R1, R2 and R3 are the reverse primers.

5’ fluorescent primers (6-Fam or VIC) of the R1, R2 and R3 primers were used during the primer extension reactions.
